# Sympathetic Denervation Ameliorates Renal Fibrosis *via* Inhibition of Cellular Senescence

**DOI:** 10.3389/fimmu.2021.823935

**Published:** 2022-01-24

**Authors:** Qian Li, Yuanjun Deng, Lele Liu, Chunjiang Zhang, Yang Cai, Tianjing Zhang, Min Han, Gang Xu

**Affiliations:** Division of Nephrology, Department of Internal Medicine, Tongji Hospital, Tongji Medical College, Huazhong University of Science and Technology, Wuhan, China

**Keywords:** sympathetic denervation, cellular senescence, neurometabolic, neuroimmune, renal fibrosis.

## Abstract

**Objective:**

Continuous overactivation of the renal sympathetic nerve is considered to be an important cause of renal fibrosis. Accumulated senescent cells in the damaged kidney have metabolic activities and secrete amounts of proinflammatory factors as part of the SASP (the senescence-associated secretory phenotype), which induce chronic inflammation and fibrosis. It is still unclear whether renal sympathetic nerves affect renal inflammation and fibrosis by regulating cellular senescence. Therefore, we hypothesize that sympathetic activation in the injured kidney induces cellular senescence, which contributes to progressive renal inflammation and fibrosis.

**Methods:**

Renal denervation was performed 2 days before the UUO (unilateral ureteral obstruction) and UIRI (unilateral ischemia-reperfusion injury) models. The effects of renal denervation on renal fibrosis and cellular senescence were observed. *In vitro*, cellular senescence was induced in renal proximal tubular epithelial cell lines (TKPTS cells) by treatment with norepinephrine (NE). The selective α_2A_-adrenergic receptor (α_2A_-AR) antagonists BRL44408 and β-arrestin2 siRNA, were administered to inhibit NE-induced cellular senescence. A significantly altered pathway was identified through immunoblotting, immunofluorescence, immunocytochemistry, and functional assays involved in mitochondrial function.

**Results:**

Renal fibrosis and cellular senescence were significantly increased in UUO and UIRI models, which were partially reversed by renal denervation. *In vitro*, NE induced epithelial cells secreting proinflammatory cytokines and promoted cell senescence by activating α_2A_-AR. Importantly, the effects of NE during cellular senescence were blocked by α_2A_-AR selective antagonist and β-arrestin2 (downstream of α_2A_-AR) siRNA.

**Conclusion:**

Renal sympathetic activation and cellular senescence are important neurometabolic and neuroimmune mechanisms in the development of renal fibrosis. Renal sympathetic neurotransmitter NE acting on the α_2A_-AR of epithelial cells promotes cellular senescence through the downstream β-arrestin2 signaling, which is a potential preventive target for renal fibrosis.

## Introduction

The incidence and prevalence of chronic kidney disease (CKD) have been rapidly increasing in recent years, especially in the elderly (1, 2). The epidemic surveys showed that the prevalence of CKD in the elderly was 3- to 13-fold higher than that in younger individuals (2). In the elderly, the presence of other diseases, such as hypertension, atherosclerosis, and diabetes, could accelerate the age-related decline of renal function (3). Thus, the elderly was more sensitive to renal injury and more likely developed to CKD (4). Similar to aging kidneys (5, 6), pathological changes of CKD included glomerular sclerosis, tubular atrophy, and interstitial fibrosis (7). These findings suggested that CKD was an “accelerated aging” phenotype (8).

Chronic senescent cell accumulation was found in the renal tissues of various CKDs (9). Premature aging deteriorates the health, life quality, and survival time of patients with CKD (10). Uremic toxins accelerated the progress of cellular senescence in CKD (8), while chronic senescent cells accumulating in aging and damaged kidneys ulteriorly drove renal fibrogenesis (9). These senescent cells underwent metabolic reprogramming and produced important proinflammatory and profibrogenic mediators as part of the SASP, which contributed to chronic inflammation and organ fibrosis (11).

The nerves innervating the kidney are composed of efferent sympathetic nerves and afferent sensory nerves (12). Similar to the cardiovascular system, the renal physiological and pathological functions are also regulated by sympathetic nerves (13, 14). It has been proved that efferent sympathetic nerve fibers are distributed in juxtaglomerular granular cells, renal tubular epithelial cells, and renal vasculature (13). In CKD patients, sympathetic excitability was improved, which contributes to CKD progression. Accumulating studies revealed that sympathetic nerve activities and tissue contents of neurotransmitter norepinephrine are increased in CKD patients and experimental animals (15, 16). Renal denervation has been proved to be an effective intervention of renal interstitial fibrogenesis in obstructive nephropathy and ischemia-reperfusion injury animal models (17, 18). Although the potential mechanisms, including disordered inflammatory response, cell cycle arrest, apoptosis-related signaling pathways, and reduced nitric oxide (NO) availability, were indicated to be related to CKD progression induced by sympathetic excitation (16, 19), the exact role and mechanism of the sympathetic pathway are still unclear.

In this study, we sought to clarify the possible mechanism of sympathetic nerves promoting renal fibrosis by inducing cellular senescence. The findings of our study have not only provided more comprehensive neurometabolic/neuroimmune mechanisms for renal fibrosis but also clarified a detailed signaling pathway involved in sympathetic nerve-induced senescence.

## Materials and Methods

### Mice and Surgical Preparation

Mice aged 8 weeks were grouped as sham-operated, UUO, UIRI, and renal denervation (DNx). UUO and UIRI surgery were performed as described previously (20, 21). Briefly, the UUO-operated group was conducted by ligating completely the left ureter with 5-0 silk sutures. The UIRI group was established in the mice subjected to clamping of the left renal pedicle for 30 min at 37°C. DNx was performed by stripping the sheath and adventitia of the left renal pedicle through the abdominal incision, as previously reported (17, 22). Briefly, after the renal artery and vein were dissected carefully from the surrounding connective tissue, the visible nerve fibers were cut off. Then the renal vessels were painted for 2 min with sterile gauzes soaked in 10% phenol anhydrous ethanol solution to exterminate the remaining nerves. Notably, the body temperature of the mice was kept between 36.6° and 37.2°C during the whole process. Mice were euthanized 10 days after UUO or 18 days after UIRI surgery. All animal procedures were performed following the Huazhong University of Science and Technology of Health guidelines.

### Cell Culture and Treatment

Mouse renal proximal tubular epithelial cell lines (TKPTS cells, American Type Culture Collection, Manassas, VA, USA) were maintained using a DMEM-F12 medium (Gibco, Carlsbad, CA, USA), which was added with 10% fetal bovine serum (Gibco). When 40% confluent, cells were serum-free for 8 hours before norepinephrine (10 nM, HY-13715B, MedChemExpress, USA) or H_2_O_2_ (100 μM, Promoter Biotechnology, China) treatment for 48 hours. In some experiments, cells were pretreated with Atipamezole (α_2_-adrenergic antagonist, HY-12380A, MedChemExpress), Butoxamine (β_2_-adrenergic antagonist, B1385, Sigma), ICI118551 (β_2_-adrenergic antagonist, HY-13951, MedChemExpress), and BRL44408 (α_2A_-adrenergic antagonist, ab120806, Abcam, Cambridge, MA, USA) an hour before norepinephrine stimulation.

### Transfection of siRNA

β-arrestin2 small interfering RNA (siRNA) was designed and synthesized by Ribobio Biotechnology (Guangzhou, China). The TKPTS cells were transfected transiently with β-arrestin2 or scrambled siRNA diluted in Lipofectamine iMAX (Thermo Fisher Scientific, USA) 24 hours before norepinephrine treatment.

### Western Blot Analysis

Protein homogenates were made from the left kidney or the cultured TKPTS cells. Western blot analyses were conducted following the manufacturer’s instructions with specific primary antibodies: fibronectin (15613-1-AP, Proteintech, Rosemont, IL, USA), College 1 (14695-1-AP, Proteintech), α-SMA (ab5694, Abcam), tyrosine hydroxylase (TH, ab112, Abcam), p53 (A3185, ABclonal Technology, China), p21 (14-6715-81, Invitrogen), γ-H2AX (#9718, Cell Signaling Technology, Danvers, MA, USA), α_2A_-AR (DF3076, Affinity Biosciences), β-arrestin2 (10171-1-AP, Proteintech), VDAC1 (55259-1-AP, Proteintech), COX IV (11242-1-AP, Proteintech), and NF-κB p65 (sc-8008, Santa Cruz Biotechnology). Anti-GAPDH (AC001, ABclonal Technology) or β-actin antibody (AC026, ABclonal Technology) was adopted for loading controls on membranes. Subsequently, the horseradish peroxidase (HRP)-conjugated secondary antibodies (Servicebio, China) were applied to combine the primary ones for 90 min at room temperature. Finally, the ECL system was used to visualize proteins, and the Image J software (NIH, USA) was applied to analyze bands.

### Quantitative Real-Time PCR (qRT-PCR)

RNA in kidney tissues or cells was isolated following the RNA extraction process of the manufacturer, which was assisted by the TRIzol (9109, TaKaRa, Japan). The cDNA was synthesized using 1 μg of RNA through a HiScript II Q RT SuperMix for qPCR kit (R222-01, Vazyme) at 50°C for 15 min and 85°C for 5 s. After using a ChamQTM Universal SYBR^®^ qPCR Master Mix kit (Q711-02, Vazyme), qRT-PCR was performed on a Roche Light 480 II system. Mouse GAPDH or β-actin was used as the housekeeping gene, and the 2-ΔΔCt method was adopted to calculate the relative quantitative evaluation of the target genes. **Table 1** shows the primer sequences.

**Table 1:** The primer sequences used for qRT-PCR.

Gene	Species	Forward primer	Reverse primer
FN	Mouse	TTCAAGTGTGATCCCCATGAAG	CAGGTCTACGGCAGTTGTCA
Col 1	Mouse	AAGAAGCACGTCTGGTTTGGAG	GGTCCATGTAGGCTACGCTGTT
α-SMA	Mouse	TCAGGGAGTAATGGTTGGAATG	CCAGAGTCCAGCACAATACCAG
p53	Mouse	CCCCTGTCATCTTTTGTCCCT	AGCTGGCAGAATAGCTTATTGAG
p21	Mouse	CAGCCATGACGAGCTGTTCT	CTTTCGGTACCTTCGCCCTC
p16	Mouse	ACATCAAGACATCGTGCGATATT	CCAGCGGTACACAAAGACCA
HMGA2	Mouse	CACATCAGCCCAGGGACAAC	TTGCTGCCTTTGGGTCTTCC
H2AX	Mouse	AGTACCTCACTGCCGAGATCC	TACTCCTGAGAGGCCTGCGA
TGF-β1	Mouse	GAGCCCGAAGCGGACTACTA	TGGTTTTCTCATAGATGGCGTTG
CTGF	Mouse	GGACACCTAAAATCGCCAAGC	ACTTAGCCCTGTATGTCTTCACA
IL-6	Mouse	TAGTCCTTCCTACCCCAATTTCC	TTGGTCCTTAGCCACTCCTTC
IL-8	Mouse	TGTTGAGCATGAAAAGCCTCTAT	AGGTCTCCCGAATTGGAAAGG
IL-1β	Mouse	GCAACTGTTCCTGAACTCAACT	ATCTTTTGGGGTCCGTCAACT
ADRA1B	Mouse	CGGACGCCAACCAACTACTT	AACACAGGACATCAACCGCTG
ADRA2A	Mouse	GTGACACTGACGCTGGTTTG	CCAGTAACCCATAACCTCGTTG
ADRB1	Mouse	CTCATCGTGGTGGGTAACGTG	ACACACAGCACATCTACCGAA
ADRB2	Mouse	GGGAACGACAGCGACTTCTT	GCCAGGACGATAACCGACAT
ADRB3	Mouse	GGCCCTCTCTAGTTCCCAG	TAGCCATCAAACCTGTTGAGC
Arrb2	Mouse	GGAGTAGACTTTGAGATTCGAGC	CTTTCTGATGATAAGCCGCACA

### Histology, Immunohistochemistry, Immunofluorescence

Mouse paraffin-embedded kidney sections (4-µm thickness) fixed in 4% paraformaldehyde were subjected to the Periodic Acid-Schiff (PAS) Stain, Sirius Red Stain, and Masson Stain according to the manufacturer’s procedures. Immunohistochemistry and immunofluorescence were performed following the manufacturer’s protocols with specific antibodies against α_2A_-AR (A2809, ABclonal Technology) and β-arrestin2 (10171-1-AP, Proteintech). Samples were exposed to peroxidase-conjugated secondary antibodies followed by incubation with hematoxylin to visualize the cell nuclei for immunohistochemistry. As for immunofluorescence, samples were incubated with fluorescence-labeled secondary antibodies (Servicebio), and nuclei were stained with 4′,6-diamidino-2-phenylindole (DAPI). Finally, representative images were acquired by using a fluorescence microscope.

### SA-β-gal, MitoTracker, and TMRE Staining

TKPTS cells cultured on plates were performed SA‐β‐gal,MitoTracker, and TMRE staining according to the guides of the manufacturer. SA‐β‐gal staining was used to test the β‐galactosidase activity (C0602, Beyotime Biotechnology, Shanghai, China) for the detection of senescence. MitoTracker deep red (9082P, Cell Signaling Technology) and Tetramethylrhodamine, Ethyl Ester, Perchlorate (TMRE, T669, Thermo Fisher Scientific) staining were adopted to measure mitochondria and mitochondrial membrane potential. TKPTS cells were incubated in MitoTracker (100 nM) or TMRE (200 nM) at 37°C against the light for 30 min before performing the fluorescence activation.

### Transmission Electron Microscopy

TKPTS cells cultured on plates were collected and prepared for assessing mitochondrial morphology using a transmission electron microscope provided by the Electron Microscope Center of Renmin Hospital of Wuhan University.

### Immunoprecipitation

Lysates containing 4 mg of total protein were made using NP40 lysis buffer (Promotor, Wuhan, China) in TKPTS cells cultured on plates and were incubated with 4 μg of an anti-β-arrestin2 antibody at 4°C overnight. Normal IgG (2729S, Cell Signaling Technology) was used as a negative control. Then Protein A/G Plus Agarose (sc-2003, Santa Cruz Biotechnology) was put into the compound for 8 hours at 4°C. After the beads were washed 3 times, the lysates were resuspended and boiled. Immunoblotting was operated as a method of Western blot.

### Statistical Analyses

Data among multiple groups were assessed by one-way ANOVA followed by Tukey’s multiple comparisons test, and data between two groups were analyzed *via* nonparametric two-tailed unpaired t-tests through the GraphPad Prism software for Windows Version 8.0 (La Jolla, CA, USA). All data were shown as means ± SEMs. P values < 0.05 mean statistical significance. *P<0.05, **P<0.01, ***P<0.001, and ****P<0.0001.

## Results

### Renal Denervation Inhibits Renal Fibrosis During UUO and UIRI

**Figure 1:**
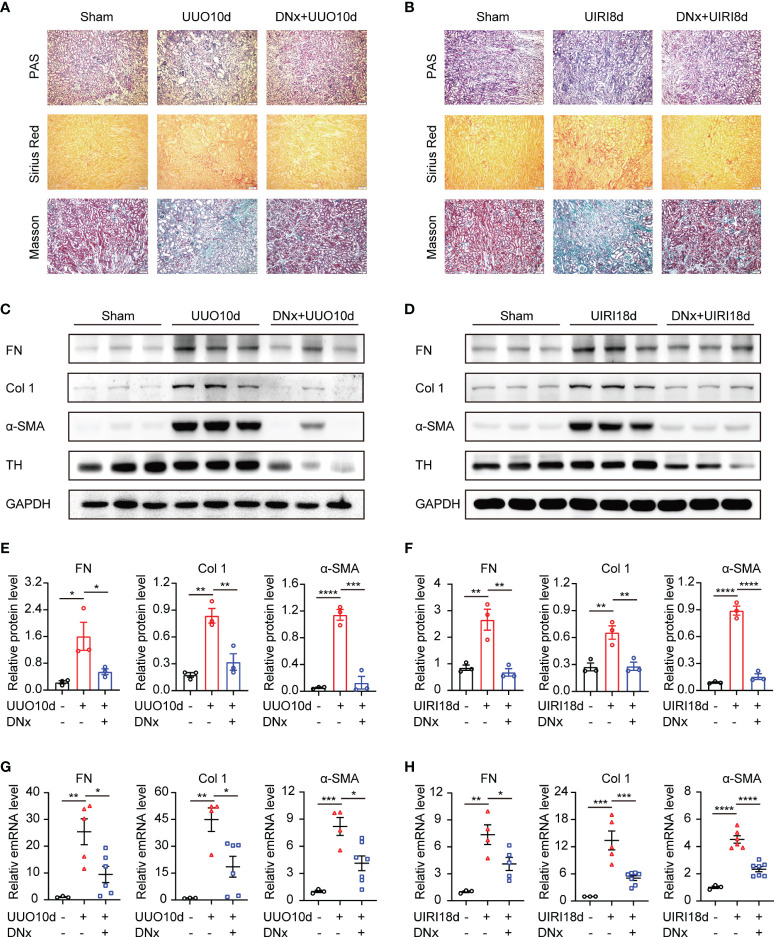
Renal denervation inhibits the fibrotic phenotype during UUO and UIRI. C57BL/6 mice were subjected to UUO or UIRI manipulation 2 days after renal denervation (DNx) or sham operation in the left kidneys. **(A, B)** Representative histology of kidney sections from sham-operated, UUO/UIRI mice, and DNx+UUO/UIRI mice stained with periodic acid–Schiff, Sirius Red, and Masson’s trichrome stain. Scale bar, 50μm. **(C, D)** Representative Western blot of fibronectin (FN), Collagen I (Col 1), α-SMA, and Tyrosine Hydroxylase (TH) in the kidney tissue of sham-operated, UUO/UIRI mice, and DNx+UUO/UIRI mice with densitometry analysis **(E, F)**. **(G, H)** Representative mRNA expressions of FN, Col 1, and α-SMA in the kidney tissue of sham-operated, UUO/UIRI mice, and DNx+UUO/UIRI mice using qRT-PCR analysis. n=3–7 in each group. *P < 0.05, **P < 0.01, ***P < 0.001, ****P < 0.0001. Bars represented means ± SEM.

To assess whether sympathetic nerves are involved in renal fibrosis, we constructed renal denervation (DNx) 2 days before UUO and UIRI. Light microscopy indicated interstitial fibrosis in the UUO-10d and UIRI-18d mice, but not in the sham-operated group, which was dramatically reduced after DNx treatment (**Figures 1A, B** **)**. The effectiveness of DNx was confirmed by a significantly diminished expression of tyrosine hydroxylase (TH), a marker of sympathetic nerve fibers (17, 18), suggesting that the model of renal denervation was successfully performed (**Figures 1C, D** **)**. By analyzing the effects of renal denervation on protein and mRNA expression, we showed that renal denervation attenuated profibrotic protein production in UUO or UIRI mice, which was measured by fibronectin (FN), collage 1 (Col 1), and α-SMA (**Figures 1C–H**). Taken together, these results indicate that sympathetic activation is closely related to renal fibrosis, and renal denervation has an obvious effect on reducing interstitial fibrosis.

### Renal Denervation Reduces Renal Senescence and Inflammation After Kidney Injury

**Figure 2:**
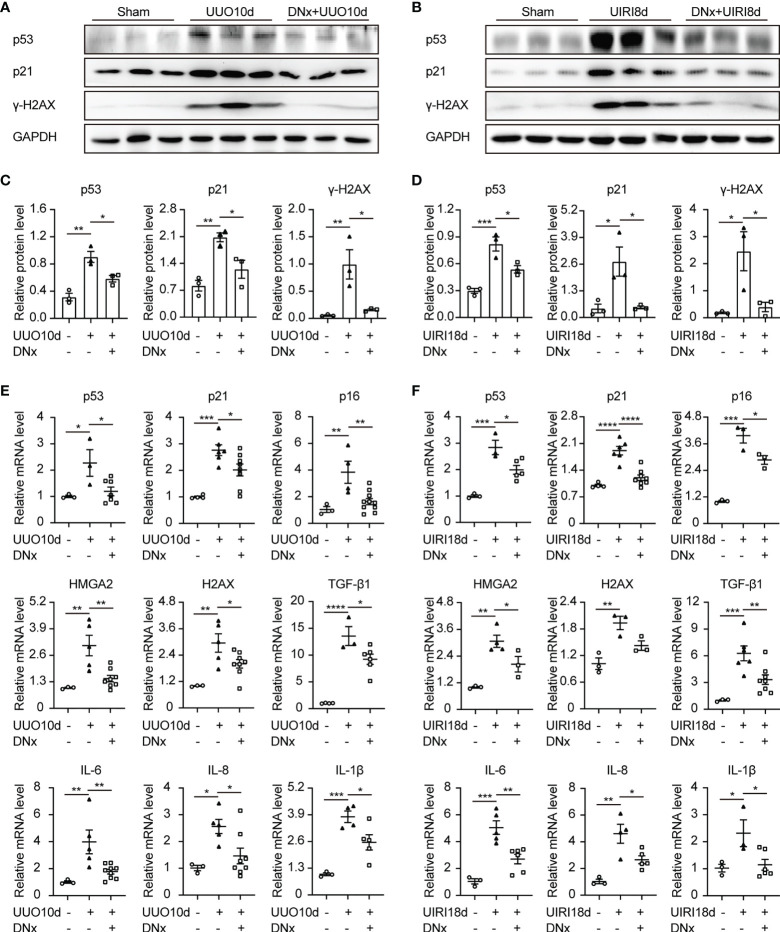
Renal denervation reduces renal senescence and the expression of proinflammatory cytokines after kidney injury. Renal denervation (DNx) or sham operation was performed 2 days before the UUO or UIRI model in the left kidneys of mice. **(A, B)** Representative Western blot of senescence-related proteins p53, p21, and γ-H2AX in sham-operated, UUO/UIRI, and DNx+UUO/UIRI kidneys with densitometry analysis **(C, D)**. **(E, F)** mRNA expressions of senescence-related markers p53, p21, p16, HMGA2, H2AX, and senescence-associated secretory phenotype (SASP) TGF-β1, IL-6, IL-8, and IL-1β in the kidney tissue of sham-operated, UUO/UIRI mice, and DNx+UUO/UIRI mice as shown by qRT-PCR analysis. n=3–10 in each group. *P < 0.05, **P < 0.01, ***P < 0.001, ****P < 0.0001. Bars represented means ± SEM.

Recent work has indicated that increasing cellular senescence acts as a key contributor to renal inflammation and fibrosis (23, 24). To analyze the effect of renal sympathetic nerves on renal senescence, we detected expressions of senescence-related proteins and senescence-associated secretory phenotype (SASP) in intact or denervated kidneys. Protein expressions of p53, p21, and γ-H2AX (senescent markers) were increased in intact kidneys induced by UUO or UIRI, whereas renal denervation significantly attenuated their expression (**Figures 2A–D**). Proinflammatory genes, as part of the SASP, are expressed by senescent cells (25). Our results demonstrated that senescence-related symbols (p53, p21, p16, HMGA2, H2AX) and inflammatory components of the SASP (TGF-β1, IL-6, IL-8, and IL-1β) were elevated in mice of UUO or UIRI, which were decreased in denervated kidneys (**Figures 2E, F**). Taken together, these data demonstrate that cellular senescence is closely related to renal inflammation and fibrosis, and renal denervation inhibits cellular senescence.

### NE Triggers Cellular Senescence and Upregulates Proinflammatory Cytokines and Profibrogenic Factors in Renal Tubular Cells

**Figure 3:**
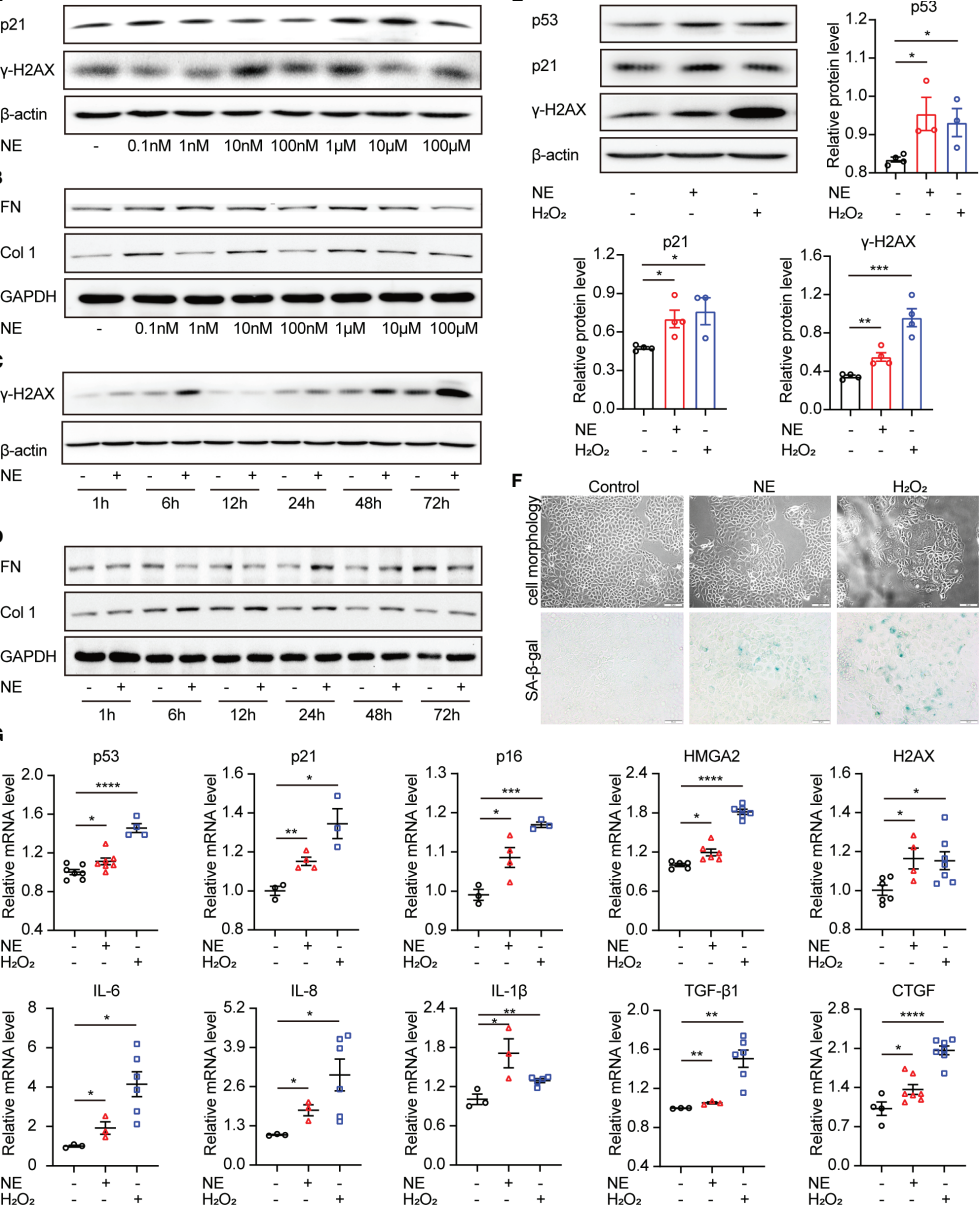
Norepinephrine contributes to cellular senescence and upregulates profibrogenic and proinflammatory cytokines in renal tubular cells. **(A, B)** Representative Western blot of p21, γ-H2AX or FN, Col 1 in TKPTS cells cultured with PBS (control) or different concentrations of NE (0.1 nM, 1 nM, 10 nM, 100 nM, 1 μM, 10 μM, and 100 μM) (n=3 in each group). **(C, D)** Representative Western blot of γ-H2AX or FN, Col 1 in TKPTS cells incubated with PBS (control) or NE at a final concentration of 10 nM for the indicated periods (1, 6, 12, 24, 48, and 72 hours) (n=3 in each group). **(E–G)** TKPTS cells were incubated with NE (10 nM) or H_2_O_2_ (100 μM) for 48 hours and were then harvested. **(E)** Representative Western blot of p53, p21, and γ-H2AX in TKPTS cells treated with PBS, NE, or H_2_O_2_ with densitometry analysis (n=3–5 in each group). **(F)** Representative images of cell morphology and SA-β-gal staining of TKPTS cells treated with PBS, NE, or H_2_O_2_ using a microscope (n=3 in each group). Scale bar=50 μm. **(G)** mRNA levels of senescence-related proteins (p53, p21, p16, HMGA2, H2AX), SASP components (IL-6, IL-8, IL-1β), and profibrotic cytokines (TGF-β1, CTGF) in each group were detected by qRT-PCR analysis (n=3–7 in each group). *P < 0.05, **P < 0.01, ***P < 0.001, ****P < 0.0001. Bars represented means ± SEM.

As a primary neurotransmitter released by the sympathetic nerve fibers, NE participates in kinds of renal physiological functions, including renal blood flow, renal tubular reabsorption of sodium and water, as well as the neural control of renal functions (26). To identify the effect of sympathetic activity on renal fibrosis and cellular senescence, TKPTS cells were utilized and treated with exogenous NE to imitate the state of sympathetic activation. As shown in **Figures 3A–D**, Western blot analysis revealed that NE markedly increased the levels of senescence-related proteins (p21, H2AX) and profibrotic proteins (FN, Col1) in a time- and concentration-dependent manner. The optimal exposure concentration and time of NE were 10 nM and 48 hours, respectively. Thus, TKPTS cells were harvested with NE (10 nM) for 48 hours in the following experiments. Notably, in the NE-treated group, the expression levels of p53, p21, and γ-H2AX were significantly increased, which was similar to the results from TKPTS cells for the indicated periods after hydrogen peroxide (H_2_O_2_) treatment [a classic stimulator of cellular senescence (27)] (**Figure 3E**). Compared with basal conditions, NE-/H_2_O_2_ treatment induced a significant increase in the enzymatic activity of SA-β-Gal (senescence-associated β-galactosidase), a hallmark of senescence (**Figure 3F**). Furthermore, RT-PCR results showed that the mRNA levels of senescence-related proteins (p53, p21, p16, HMGA2, H2AX), SASP components (IL-6, IL-8, IL-1β, TGF-β1), and profibrogenic growth factor CTGF were increased by NE stimulation, which was consistent with the results in the H_2_O_2_ group (**Figure 3G**). These data suggest that NE contributes to renal tubular cell senescence and upregulates proinflammatory and profibrogenic components.

### The α_2A_-Adrenergic Receptor Is Responsible for NE-Induced Cellular Senescence

**Figure 4:**
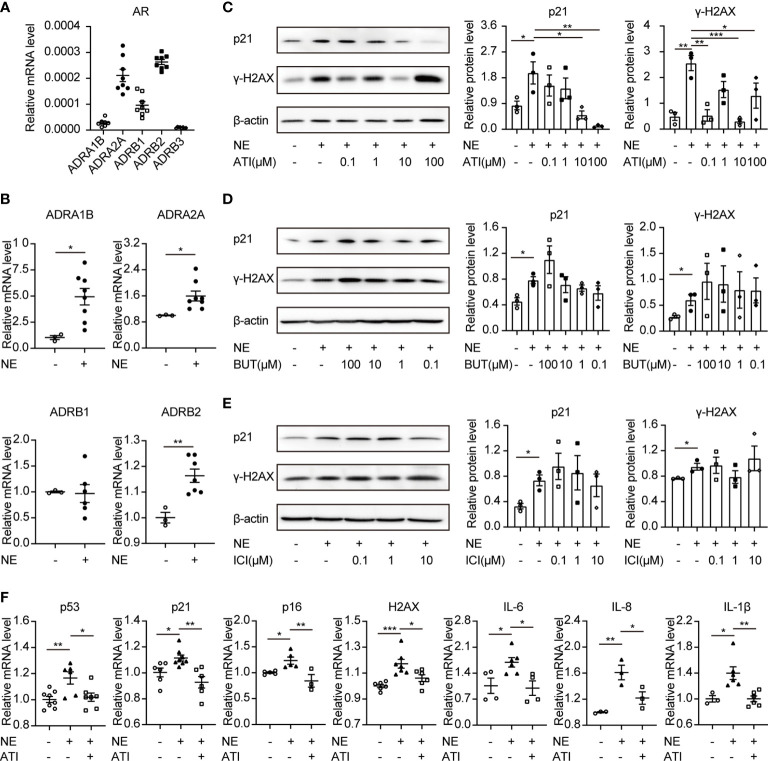
Atipamezole (α_2_-adrenergic antagonist) reverses cellular senescence induced by NE in renal tubular cells. TKPTS cells were incubated with NE (10 nM) for 48 hours after pretreatment with adrenergic antagonists or PBS for an hour. **(A, B)** mRNA expressions of ADRA1B, ADRA2A, ADRB1, ADRB2, and ADRB3 in PBS (control) or NE-treated TKPTS cells as shown by qRT-PCR analysis (n=3–8 in each group). **(C)** Representative Western blotting of p21 and γ-H2AX in TKPTS cells treated with PBS, NE, and NE+ATI (Atipamezole, 100 nM, 1 μM, 10 μM, and 100 μM) with densitometry analysis (n=3 in each group). **(D, E)** Representative Western blotting of p21 and γ-H2AX in TKPTS cells incubated with PBS, NE, and NE+BUT (Butoxamine)/ICI (ICI118551) (two β_2_-adrenergic antagonists, 100 μM, 10 μM, 1 μM, and 100 nM) with densitometry analysis, respectively (n=3 in each group). **(F)** mRNA expressions of p53, p21, p16, H2AX, IL-6, IL-8, and IL-1β in TKPTS cells cultured with PBS, NE, and NE+ATI (10 μM) as displayed by qRT-PCR analysis (n=3–8 in each group). *P < 0.05, **P < 0.01, ***P < 0.001. Bars represented means ± SEM.

NE participates in signal transductions through the adrenergic receptors (ARs), a member of the GPCR families. ARs consist of two α-AR (α_1_-, α_2_-AR) and three β-AR (β_1_-, β_2_-, β_3_-AR) (28). Thus, we investigated the roles of ARs in cellular senescence using their antagonists. Firstly, cDNA was used to determine the mRNA expression levels of five AR coding genes (ADRA1B, ADRA2A, ADRB1, ADRB2, and ADRB3) (29). Results showed that ADRB2 (coding β_2_-AR) and ADRA2A (coding α_2A_-AR) were enriched in TKPTS cells (**Figure 4A**). Furthermore, the expression of ADRB2 and ADRA2A were upregulated after NE treatment (**Figure 4B**). To identify which adrenergic receptor mediates renal tubular epithelial cell senescence induced by NE, we pretreated TKPTS cells with β_2_-AR and α_2_-AR adrenergic antagonists, respectively. Forty-eight hours after NE stimulation, cells pretreated with α_2_-AR antagonist Atipamezole (ATI) at the concentrations of 10–100 μM showed significant reductions in p21 and γ-H2AX protein levels (**Figure 4C**), whereas treatment with the two β_2_-AR antagonists (Butoxamine and ICI118551) showed no reductions in p21 and γ-H2AX protein expressions (**Figures 4D, E** **)**. Consistent with the results from Western blot, RT-PCR analysis also showed that TKPTS cells pretreated with α_2_-AR antagonist ATI significantly reduced the mRNA levels of senescence-related proteins (p53, p21, p16, HMGA2, H2AX) and proinflammatory cytokines (IL-6, IL-8, IL-1β) (**Figure 4F**). These data suggest that NE induces cellular senescence through α_2_-AR, and pretreatment with the α_2_-adrenergic antagonist suppresses the senescence phenotype and inflammatory response induced by NE.

**Figure 5:**
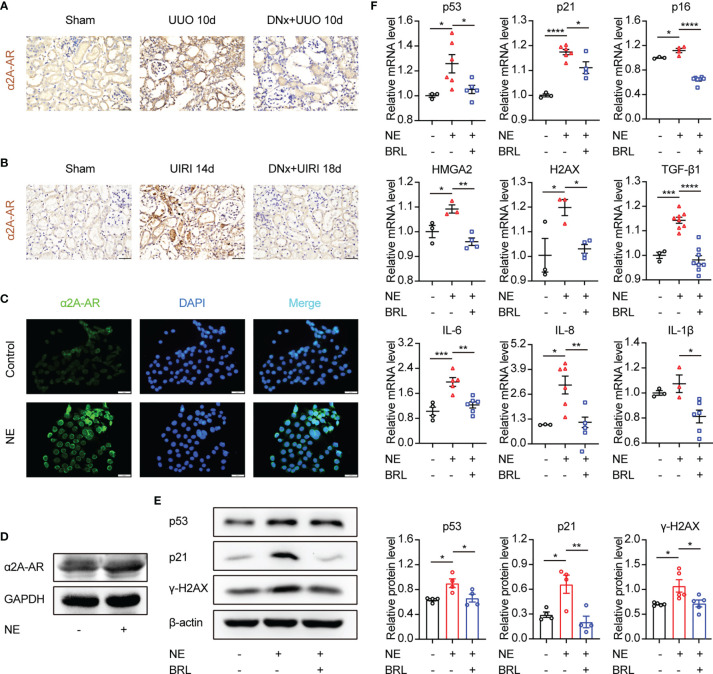
The α_2A_-adrenergic receptor is responsible for NE-induced tubular epithelial cell senescence. **(A, B)** Renal denervation (DNx) or sham operation was performed 2 days before UUO or UIRI in the left kidneys of mice. Representative images of immunohistochemistry for α_2A_-adrenergic receptors (α_2A_-AR) in sham-operated, UUO/UIRI, mice and DNx+UUO/UIRI mice. Scale bar, 20μm. **(C, D)** After starving for 8 hours, TKPTS cells were cultured with PBS or NE (10 nM) for 48 hours. α_2A_-AR expressions were tested by immunofluorescence **(C)** (Scale bar, 20 μm) and Western Blot **(D)** (n=3 in each group). **(E, F)** After pretreatment with PBS or α_2A_-adrenergic antagonist (BRL4408, BRL, 10 μM) for 1 hour, TKPTS cells were incubated with NE (10 nM) for 48 hours. **(E)** Representative Western blotting of p53, p21, and γ-H2AX in each group with densitometry analysis (n=4-5). **(F)** mRNA expressions of p53, p21, p16, HMGA2, H2AX, TGF-β1, IL-6, IL-8, and IL-1β were analyzed using qRT-PCR analysis (n=3–8 in each group). *P < 0.05, **P < 0.01, ***P < 0.001, ****P < 0.0001. Bars represented means ± SEM.

α_2_-AR consists of α_2A_-AR, α_2B_-AR, and α_2C_-AR subtypes. Kable JW et al. demonstrated that the classical pharmacological actions of α_2_-AR were mainly mediated by the α_2A_-AR subtype, including antihypertensive, sedative, analgesic, and so on (30). Thus, in the present study, α_2A_-AR expression in renal tissue and TKPTS cells was further examined. Results showed that α_2A_-AR was significantly increased in kidney sections from UUO or UIRI mice, whereas renal denervation markedly lessened α_2A_-AR expression (**Figures 5A, B** **)**. Moreover, compared with the control group, the expression of α_2A_-AR was significantly increased in cells treated with NE (**Figures 5C, D** **)**. To test whether α_2A_-AR contributes to tubular epithelial cell senescence induced by NE, selective α_2A_-AR antagonist BRL44408 (BRL, 10 μM) was applied to TKPTS before NE stimulation. Western blot analysis demonstrated that BRL pretreatment significantly decreased the expression of p53, p21, and γ-H2AX compared with that in the NE group (**Figure 5E**). What is more, BRL pretreatment abolished NE-induced increase in the mRNA expressions of senescence-related proteins (p53, p21, p16, HMGA2, H2AX) and SASP components (TGF-β1, IL-6, IL-8, and IL-1β) (**Figure 5F**). Taken together, these data suggest that α_2A_-AR plays a crucial role in tubular epithelial cell senescence and is closely associated with inflammatory cytokine expression induced by NE.

### α_2A_-AR/β-Arrestin2 Signaling Contributes to Cellular Senescence Induced by NE

**Figure 6:**
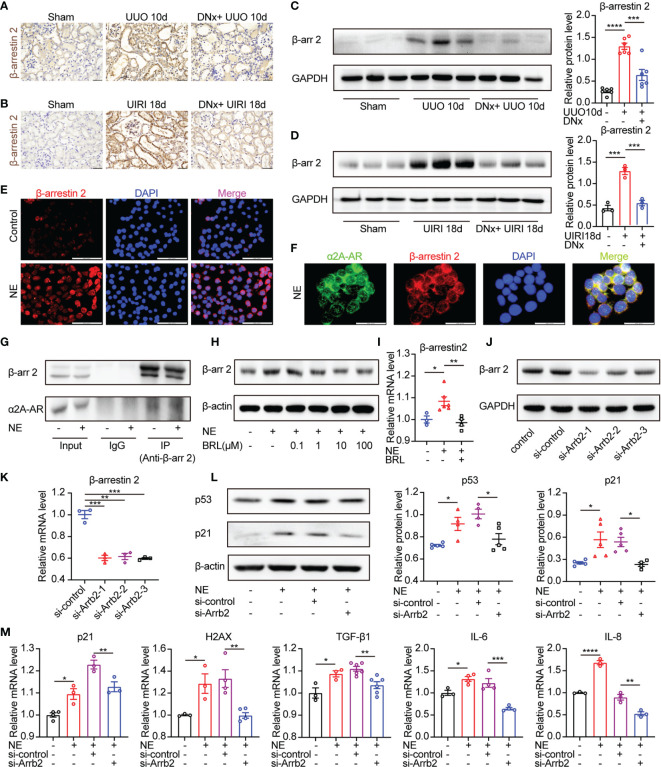
β-arrestin2 is a target of α_2A_-AR and knockdown of β-arrestin2 ameliorates NE-induced tubular cellular senescence. **(A, B)** Renal denervation (DNx) or sham operation was performed 2 days before UUO or UIRI in the left kidneys of mice. Representative images of immunohistochemistry for β-arrestin2 in sham-operated, UUO/UIRI mice, and DNx+UUO/UIRI mice. Scale bar, 20 μm. **(C, D)** Representative Western blotting of β-arrestin2 (β-arr2) in sham-operated, UUO/UIRI, and DNx+UUO/UIRI kidneys with densitometry analysis (n=3–6 in each group). **(E–G)** TKPTS cells were cultured with PBS or NE (10 nM) for 48 hours. **(E)** Representative images of immunofluorescence for β-arrestin2 in each group. Scale bar, 20μm. **(F)** Cells were double-labeled with α_2A_-AR (green) and β-arrestin2 (red), and cell nuclei were enhanced by staining with DAPI (blue). Colocalization of α_2A_-AR (green), and β-arrestin2 (red) was visualized as yellow in merged images. Scale bar, 20μm. **(G)** Interactions between β-arrestin2 and α_2A_-AR were examined by immunoprecipitation (IP) with an anti-β-arrestin2 antibody, followed by immunoblotting (IB) with anti-α_2A_-AR antibodies. **(H, I)** After an hour of pretreatment with PBS or BRL4408, TKPTS cells were stimulated with NE (10 nM) for 48 hours. **(H)** Representative Western blotting of β-arrestin2 (β-arr2) in TKPTS cells treated with PBS, NE, and NE+BRL (100 nM, 1 μM, 10 μM, and 100 μM) (n=3 in each group). **(I)** mRNA expression of β-arrestin2 in PBS, NE, and NE+BRL (10 μM)-treated TKPTS cells as measured by qRT-PCR analysis (n=3–6 in each group). **(J, K)** TKPTS cells were transfected with scrambled siRNA or three β-arrestin2 siRNAs (si-Arrb2-1, si-Arrb2-2, or si-Arrb2-3) for 48 hours. β-arrestin2 (β-arr2) expression was examined by Western blot analysis **(J)** and qRT-PCR analysis **(K)** (n=3 in each group). **(L, M)** TKPTS cells were transfected with scrambled siRNA (si-control) or β-arrestin2 siRNA (si-Arrb2) 24 hours before NE (10 nM) treatment. Representative Western blotting of p53 and p21 in each group with densitometry analysis **(L)** (n=4–5 in each group). mRNA expressions of p21, H2AX, TGF-β1, IL-6, and IL-8 were tested by qRT-PCR analysis **(M)** (n=3–6 in each group). *P < 0.05, **P < 0.01, ***P < 0.001, ****P < 0.0001. Bars represented means ± SEM.

We next explored the signaling pathway downstream of the α_2A_-AR that promotes cellular senescence. α_2A_-AR belongs to G protein-coupled receptors (GPCRs). Besides the canonical Gs-cAMP pathway, the GPCR/β-arrestin2 pathway was closely related to organ fibrosis, including liver fibrosis, pulmonary fibrosis, myocardial fibrosis, and renal fibrosis (31–33). In the current study, results showed that the expression of β-arrestin2 was substantially increased on the kidney sections of mice with UUO or UIRI models compared with the sham-operated group as measured by immunohistochemistry and Western blotting (**Figures 6A–D**). On the other hand, renal denervation lessened the expression of β-arrestin2 compared with the UUO or UIRI group. To further demonstrate the direct effects of the α_2A_-AR/β-arrestin2 pathway on epithelial cells, we analyzed TKPTS cells *in vitro*. Immunofluorescence staining showed that β-arrestin2 was substantially increased in the presence of NE compared with control (**Figure 6E**). Furthermore, we observed the direct colocalization of α_2A_-AR and β-arrestin2, which was confirmed by analyzing protein–protein interaction using co-immunoprecipitation (**Figures 6F, G** **)**. Besides, TKPTS pretreated with selective α_2A_-AR antagonist BRL44408 (BRL) relieved β-arrestin2 expression using Western blot and qRT-PCR (**Figures 6H, I** **)**, illustrating the direct relationship between β-arrestin2 and α_2A_-AR. To further illustrate the role of β-arrestin2 on NE-mediated cellular senescence in TKPTS cells, siRNAs (small interfering RNAs) were used to specifically knock down β-arrestin2. As shown in **Figures 6J, K**, si-Arrb2-1 exhibited the most inhibitory effect on β-arrestin2. Thus, si-Arrb2-1 was used for subsequent experiments. Compared with the NE group, si-Arrb2 restrained the expression of p53 and p21 (**Figure 6L**). Consistently, the mRNA levels of p21, H2AX, TGF-β1, IL-6, and IL-8 were also prevented by si-Arrb2 pretreatment (**Figure 6M**). These results indicate that NE activates α_2A_-AR/β-arrestin2 signaling to induce cellular senescence and proinflammatory responses, and blockade of β-arrestin2 inhibits this process.

### α_2A_-AR/β-Arrestin2/NF-κB Signaling Is Involved in Mitochondrial Dysfunction and Cellular Senescence

**Figure 7:**
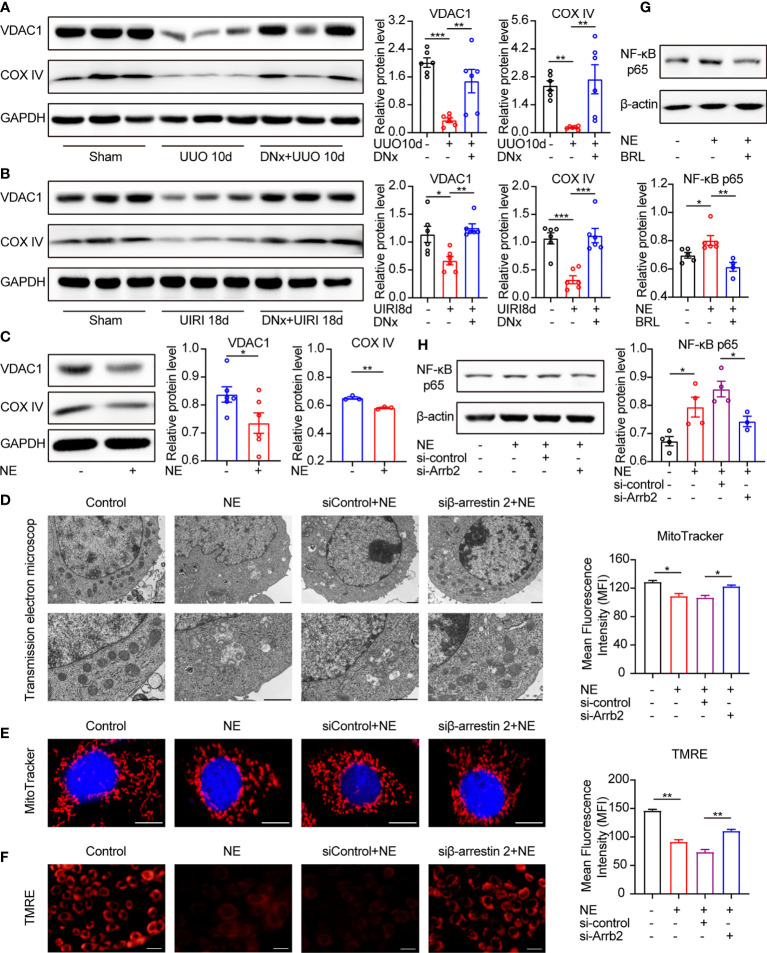
α_2A_-AR/β-arrestin2/NF-κB signaling contributes to mitochondrial dysfunction and cellular senescence. **(A, B)** Renal denervation (DNx) or sham operation was performed 2 days before UUO or UIRI in the left kidneys of mice. Representative Western blotting of VDAC1 and COX IV in each group with densitometry analysis (n=6). **(C)** Representative Western blotting of VDAC1 and COX IV in TKPTS cells treated with PBS or NE (10 nM) for 48 hours with densitometry analysis (n=3–6 in each group). **(D–F)** TKPTS cells were transfected with scrambled siRNA (si-control) or β-arrestin2 siRNA (si-Arrb2) for 24 hours and then incubated with NE (10 nM) for 48 hours. Mitochondrial ultrastructure was observed by transmission electron microscope **(D)**. Scale bar, 1 μm. Mitochondrial mass was tested by MitoTracker deep red probe (100nM) staining **(E)**. Scale bar, 50 μm. Mitochondrial membrane potential was examined by TMRE fluorescent dye (200 nM) **(F)**. Scale bar, 20 μm. **(G)** Representative Western blotting of NF-κB p65 in TKPTS cells incubated with PBS, NE, and NE+BRL (10 μM) with densitometry analysis (n=4–6 in each group). **(H)** Representative Western blotting of NF-κB p65 in TKPTS cells of PBS, NE, and NE+si-control, NE+si-Arrb2 with densitometry analysis (n=3-4 in each group). *P < 0.05, **P < 0.01, ***P < 0.001. Bars represented means ± SEM.

Considering that the mitochondria status has been closely associated with senescence and aging phenotypes (34), we aimed to determine the response of mitochondria under NE stimulation. We used the voltage-dependent anion channel 1 (VDAC1) and cytochrome c oxidase subunit IV (COX IV), markers representing the mitochondrial outer membrane and the mitochondrial inner membrane, respectively, to represent the mitochondria content (35). VDAC1 and COX IV protein levels were reduced in UUO or UIRI kidneys, whereas renal denervation could rescue their expressions (**Figures 7A, B** **)**. Besides, NE-treated TKPTS cells showed decreased expression of VDAC1 and COX IV (**Figure 7C**). These data suggest that sympathetic activation leads to mitochondrial dysfunction.

Next, we tested whether β-arrestin2 participated in NE-induced mitochondrial dysfunction. As shown in **Figure 7D**, in the presence of NE, abnormal mitochondrial morphology occurred, including swelled mitochondria and irregular and fragmented mitochondrial crest. However, pretreatment with β-arrestin2 siRNA markedly ameliorated the abnormal morphology of mitochondria using transmission electron microscopy. This result was further confirmed by MitoTracker deep red staining and TMRE, which showed that the application of β-arrestin2 siRNA meliorated mitochondrial mass (**Figure 7E**) and mitochondrial membrane potential (**Figure 7F**) in TKPTS cells. Together, these results indicate that β-arrestin2 is exactly involved in NE-induced mitochondrial dysfunction.

NF-κB signaling was reported to play important roles in mitochondrial function, and using NF-κB inhibitors improved the mitochondrial function (36). Moreover, there has been intensive research using models convincingly demonstrating that NF-κB signaling promotes SASP expression in cellular senescence (37, 38). Thus, we hypothesized that NF-κB is related to NE/α_2A_-AR/β-arrestin2 mediated senescence. As shown by our results, pretreatment with selective α_2A_-AR antagonist BRL44408 (BRL, 10μM) declined the protein expression of NF-κB p65 compared with the NE group (**Figure 7G**). Similarly, NF-κB p65 was higher in NE-treated cells as compared with the control group, which was significantly blocked by β-arrestin2 siRNA (**Figure 7H**). These results illustrate that NF-κB is downstream of α_2A_-AR/β-arrestin2 signaling in the NE-induced mitochondrial function, which contributes to cellular senescence and inflammatory responses.

## Discussion

In the present study, we have demonstrated that renal denervation significantly improved renal fibrosis and cellular senescence in UUO and UIRI mice. Likewise, in our experiments *in vitro*, NE stimulation could upregulate profibrotic proteins and proinflammatory cytokines in TKPTS cells accompanied by the activation of α_2A_-AR/β-arrestin2 signaling.

In previous studies, evidence proved that renal denervation improved physiological changes. Renal sympathetic denervation significantly lessened the damage on podocyte and albuminuria in the rat cardiorenal syndrome model and the diabetic nephropathy model (39, 40). In patients with resistant hypertension, sympathetic renal denervation reduced the incidence of albuminuria and renal resistive index (41, 42). The glomerular filtration rate (GFR) and kidney weight were also improved by renal denervation in the IRI-induced renal fibrosis (18).

Furthermore, neurogenic norepinephrine signaling has been shown to be responsible for kidney inflammation and fibrosis. In UUO mice, norepinephrine supplement after renal denervation increased the levels of inflammatory molecules and infiltration of leukocytes (17). The senescent cell is one of the important resources of these inflammatory molecules. They remain metabolically active and secrete cytokines, chemokines, and growth factors (9). Senescent cells produce SASP consisting of abundant proinflammatory cytokines such as IL-6, IL-8, and IL-1β, which are considered to be important contributors to the proinflammatory phenotype (43). IL-6 disrupted the balance of Th17/Treg differentiated from CD4^+^ T cells, which pathologically exacerbated the development of inflammatory events (44). IL-8 (or CXCL8), a chemokine, initiated the inflammation process *via* recruiting and activating neutrophils (45). IL-1β mediated NLRP3 inflammasome-related inflammatory cascade, causing the persistent inflammatory response (46). In the present study, we showed that NE-induced tubular senescence was involved in renal inflammation and fibrosis *via* the production of inflammatory cytokines IL-6, IL-8, and IL-1β. Therefore, our data suggested that cellular senescence is accurately responsible for renal fibrosis under the control of sympathetic nerves.

The main characteristic of cellular senescence consists of cell cycle arrest, SASP generation, and metabolic dysfunction. The p53/p21^CIP1^ and p16^INK4a^/Rb tumor suppressor networks are involved in the development of cell cycle arrest (11, 47). In this study, we found that the increased expressions of p53 and p21 in TKPTS cells treated with NE could be partially reversed by either α_2A_-AR blockade or β-arrestin2 siRNA, indicating that NE/α_2A_-AR/β-arrestin2 signaling was an upstream regulator of p53/p21 pathways in this process. A previous report has indicated that renal norepinephrine signals contributed to renal interstitial fibrosis through α_2A_-AR and α_2C_-AR in mice with obstructive nephropathy (17). Our data further showed that α_2A_-AR was rich in renal proximal tubule cells, and blockade of α_2A_-AR prevented NE-induced cellular senescence, suggesting that NE promoted cellular senescence mainly through the α_2A_-AR subtype (**Figure 5**). Moreover, our data showed that β-arrestin2 was a target of α_2A_-AR in NE-induced cellular senescence. Knockdown of β-arrestin2 reduced senescent markers, suggesting that β-arrestin2 was critically involved in NE-induced cellular senescence (**Figure 6**).

It was reported that p38/NF-κB signaling, STING/TBK1/NF-κB signaling, JAK/STAT/NF-κB pathway, and other signaling participated in SASP production (48). We proved that the NE/α_2A_-AR/β-arrestin2 pathway promoted the expression of SASP *via* NF-κB signaling, and it was supported by data that NF-κB p65 was increased by treatment with NE but attenuated after pretreatment with the selective α_2A_-AR antagonist and β-arrestin2 siRNA. In addition, we found that mitochondrial dysfunction was also involved in tubular cell senescence induced by NE/α_2A_-AR/β-arrestin2 signaling. Proximal tubular epithelial cells are rich in mitochondria (49). Damaged mitochondria generated reactive oxygen species (ROS) and led to a cell growth arrest *via* inducing the DNA damage response (DDR), thus contributing to cellular senescence (50). In this study, sympathetic activation induces mitochondrial disorders, including the reduction of the mitochondrial membrane proteins (VADC1 and COX IV), the increase in abnormal mitochondrial morphology (swollen mitochondria and fractured mitochondrial crest), and the vanishment of the mitochondrial membrane potential (**Figure 7**). Closed or lost VDAC1 destroyed the integrity of the mitochondrial outer membranes (51), inhibited mitochondrial respiration, and exacerbated mitochondrial division (52). Besides, the lack of electron transport chain proteins, such as the cytochrome c oxidase family (like COX IV) (53), led to electron leakage, which produced ROS-like superoxide free radicals to trigger cellular senescence (54). Partial ablation of β-arrestin2 by siRNA ameliorated mitochondrial disorders, illustrating that β-arrestin2 contributes to mitochondrial dysfunction (**Figure 7D-F**). In a previous study, β-arrestin2 knockout reduced ROS production through the downregulation of NADPH oxidase 4 (NOX4) in the mice model of hepatic fibrosis (55). Besides, both β-arrestin1 and β-arrestin2 enhanced the production of mitochondrial ROS and superoxide in cardiac fibroblasts isolated from failing adult human left ventricles (56). Based on these studies, we speculate that NE/α_2A_-AR/β-arrestin2 is one of the upstream regulators of mitochondrial ROS, which then triggers cellular senescence. Furthermore, NF-κB signaling can aggravate mitochondrial disorders (36), suggesting that NF-κB/mitochondrial dysfunction may be the downstream signaling in NE/α_2A_-AR/β-arrestin2-induced cellular senescence.

It is noteworthy that mitochondrial dysfunction can also contribute to cell apoptosis. The mechanism of mitochondria-induced apoptosis is through increased mitochondrial outer membrane permeabilization, which led to the release of apoptogenic proteins (such as cytochrome c, apoptosis-inducing factor, etc.) to the cytosol and triggered cell apoptosis (57). VDAC1 is the most abundant protein of the mitochondrial outer membrane and is closely associated with cell apoptosis (58). Some studies reported that upregulation of VDAC1 promoted cells apoptosis (59–61). However, in the present study, VDAC1 decreased both in our *in vivo* and *in vitro* experiments (**Figure 7A–C**), suggesting that sympathetic nerves-induced mitochondrial dysfunction contributed to cellular senescence, instead of apoptosis.

In conclusion, this study provides important neurometabolic and neuroimmune mechanisms in the development of renal fibrosis. Renal sympathetic activation triggers tubular senescence, which secretes SASP and promotes renal inflammation and fibrosis. Meanwhile, our work demonstrates that NE-induced tubular cellular senescence is at least partially regulated by α_2A_-AR/β-arrestin2/NF-κB signaling modules. Inhibiting the actions of α_2A_-AR or β-arrestin2, alone or in combination, might provide a potential therapeutic strategy to prevent the progression of renal fibrosis.

## Data Availability Statement

The original contributions presented in the study are included in the article/supplementary files. Further inquiries can be directed to the corresponding authors.

## Ethics Statement

The animal study was reviewed and approved by the Animal Ethics Committee at the Huazhong University of Science and Technology.

## Author Contributions

MH and GX designed the study. QL and YD prepared the main manuscript text. QL, YD, and LL performed the experiments. CZ, YC, and TZ contributed to experimental data analysis. MH and GX gave crucial guidance to the study and manuscript revision. All authors participated in reviewing the manuscript and approved the final manuscript.

## Funding

Grant supports: the National Natural Science Foundation of China (81770686, 81970591, 82000656, and 82000658).

## Conflict of Interest

The authors declare that the research was conducted in the absence of any commercial or financial relationships that could be construed as a potential conflict of interest.

## Publisher’s Note

All claims expressed in this article are solely those of the authors and do not necessarily represent those of their affiliated organizations, or those of the publisher, the editors and the reviewers. Any product that may be evaluated in this article, or claim that may be made by its manufacturer, is not guaranteed or endorsed by the publisher.
